# Supplementary material for: Suppression of ferroptosis by vitamin A or radical-trapping antioxidants is essential for neuronal development
Source: Nat Commun. 2024 Sep 1;15:7611. doi: 10.1038/s41467-024-51996-1 (PMC11366759; doi:10.1038/s41467-024-51996-1)
Supplement: Supplementary file 1 — Supplementary Information [file 41467_2024_51996_MOESM1_ESM.pdf]

Supplementary Figures for

Suppression of ferroptosis by vitamin A or radical-trapping antioxidants is essential for neuronal development

Juliane Tschuck[1,§], Vidya Padmanabhan Nair[2,§], Ana Galhoz[3,4], Carole Zaratiegui[5], Hin-Man Tai[2], Gabriele Ciceri[6], Ina Rothenaigner[1], Jason Tchieu[6,7], Brent R. Stockwell[8], Lorenz Studer[6], Daphne S. Cabianca[5], Michael P. Menden[3,9], Michelle Vincendeau[2,10,#,*], and Kamyar Hadian[1,#,*]

[1] Research Unit Signaling and Translation, Helmholtz Zentrum München, Neuherberg, Germany

[2] Endogenous Retrovirus Group, Institute of Virology, Helmholtz Zentrum München, Neuherberg, Germany

[3] Computational Health Center, Helmholtz Zentrum München, Neuherberg, Germany

[4] Department of Biology, Ludwig-Maximilians University Munich, Munich, Germany

[5] Institute of Functional Epigenetics, Helmholtz Zentrum München, Neuherberg, Germany

[6] Developmental Biology and Center for Stem Cell Biology, Memorial Sloan Kettering Cancer Center, New York, NY, USA.

[7] Present address: UC Department of Pediatrics, Division of Developmental Biology, Cincinnati Children's Hospital Medical, Cincinnati, OH, USA.

[8] Department of Biological Sciences, Department of Chemistry, Herbert Irving Comprehensive Cancer Center, Irving Institute for Cancer Dynamics, Columbia University, New York, NY, USA.

[9] Department of Biochemistry and Pharmacology, University of Melbourne, Parkville Victoria, Australia

[10] Technical University of Munich, Institute of Virology, School of Medicine, Munich, Germany

[§] These authors contributed equally. The order can be interchanged.

[#] These authors jointly supervised this work. The order can be interchanged.

[*] Correspondence:

- Kamyar Hadian: kamyar.hadian@helmholtz-muenchen.de, and
- Michelle Vincendeau: michelle.vincendeau@helmholtz-muenchen.de

Supplementary Figure 1

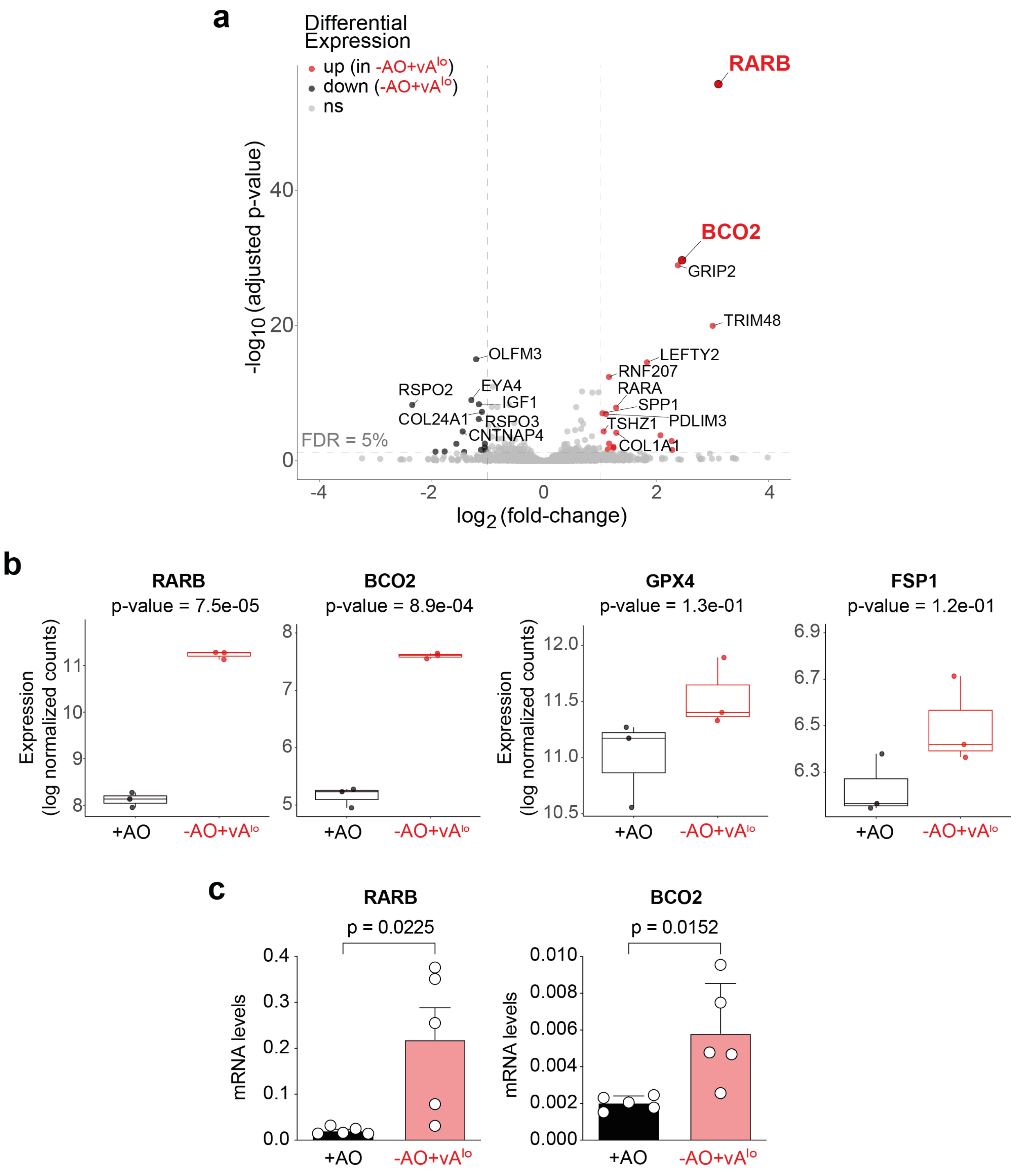

Supplementary Figure 1. RNAseq analysis of immature cortical neurons generated with or without antioxidants. a, Volcano plot of total RNA sequencing (RNA-seq) from immature cortical neurons (day 20) generated with antioxidants (+AO) or without antioxidants (-AO+vA^{lo}). A total of 33 genes were found significantly expressed (18 up-regulated and 15 down-regulated), under FDR = 5% and $|log_2FC| > 1$. The remaining genes (20,117) were classified as non-significant (*ns*) and colored in grey. The most significant genes are labelled, and genes RARB and BCO2 are highlighted. **b**, Box plot depicting expression of Retinoic Acid Receptor B (RARB), beta-carotene oxygenase 2 (BCO2), GPX4 and FSP1 in total RNA-seq of immature cortical neurons. Data are mean ± SD of n = 3 biologically independent replicates. P-values between biological conditions were computed using Student's T-Test, via *stat_compare_means()* function. **c**, Validation using quantitative RT-PCR (n = 5 biologically independent replicates). Data are mean ± SD; unpaired t-test, two-tailed.

Supplementary Figure 2

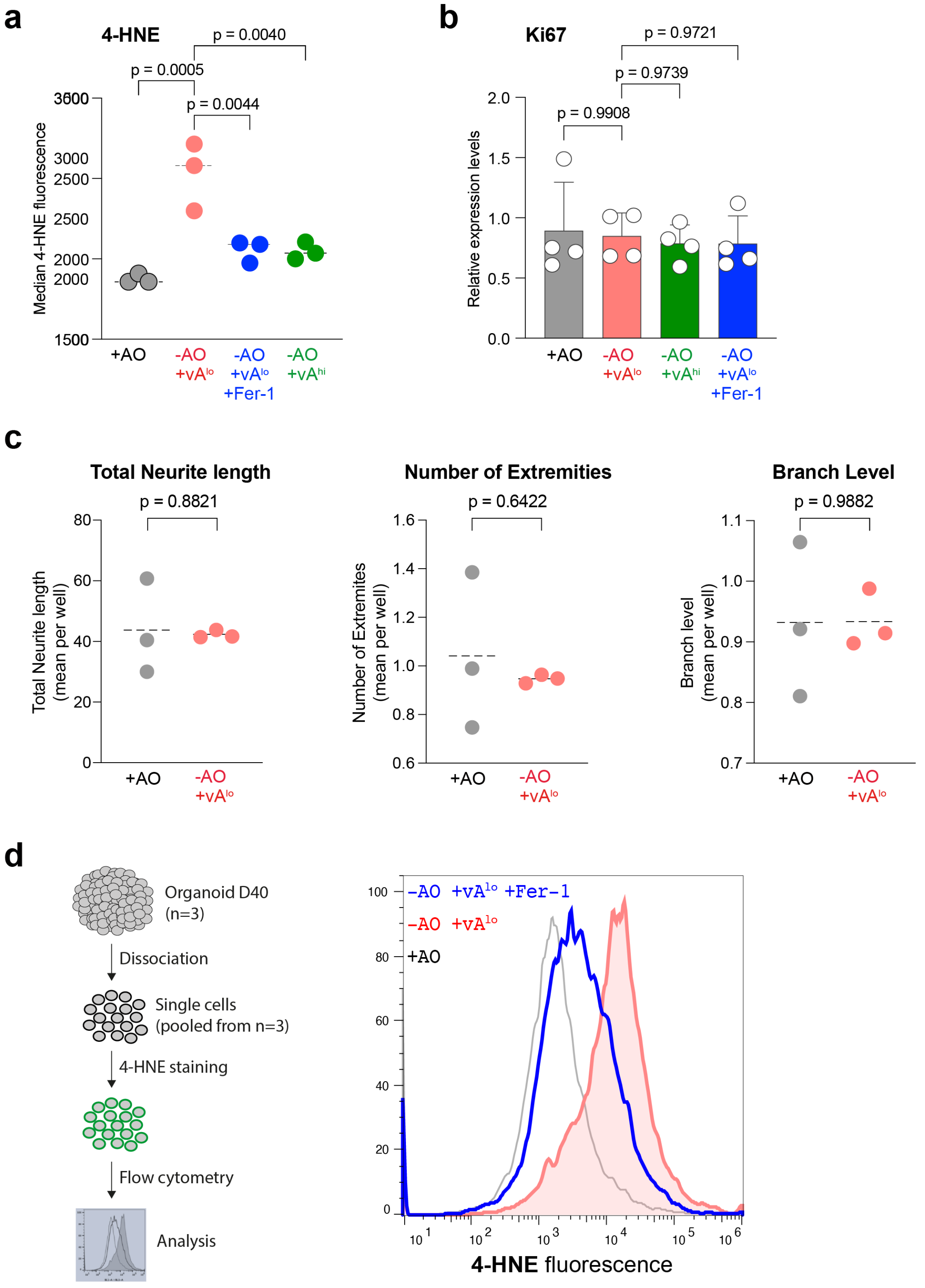

Supplementary Figure 2. Analysis of neuronal differentiation in the absence of antioxidant protection a, Quantification of 4-HNE-stained differentiated neurons at day 20 with antioxidants (+AO) or without antioxidants (-AO+vA^{lo}) as well as supplementation with Fer-1 or ATRA using flow cytometry. Data are median fluorescence of *n* = 3 biologically independent replicates; one-way ANOVA with Tukey's test. **b**, Ki67 mRNA levels of differentiated neurons at day 20 with antioxidants (+AO) or without antioxidants (-AO+vA^{lo}) as well as supplementation with Fer-1 or ATRA. Data are mean ± SD of *n* = 4 biologically independent replicates; one-way ANOVA with Tukey's test. **c**, Quantification of morphological changes of cortical neurons upon differentiation with (+AO) or without antioxidants (-AO+vA^{lo})) using high-content Image analysis. Data are mean ± SD of *n* = 3 biologically independent replicates consisting of 2 technical replicates; unpaired t-test, two-tailed. **d**, Separation of day-40 organoids (with antioxidants (+AO) or without antioxidants (-AO+vA^{lo}) as well as supplementation with Fer-1) in single cells (pool of 3 independent organoids) and subsequent 4-HNE staining and flow cytometry analysis.

Supplementary Figure 3

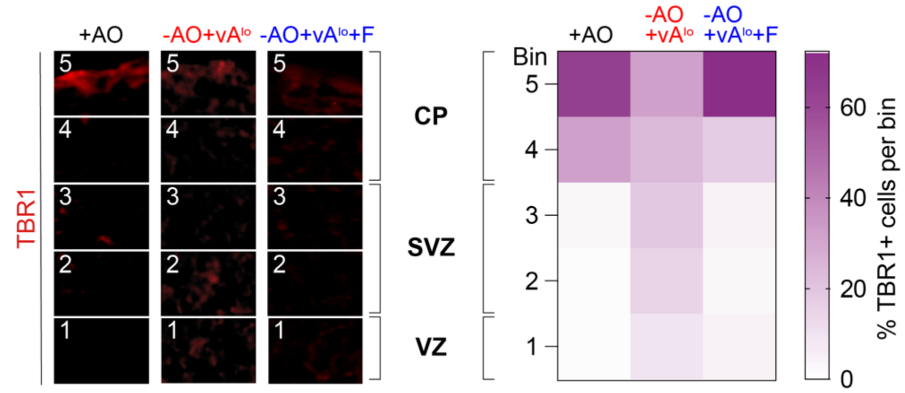

Supplementary Fig 3. Inhibition of ferroptosis is critical for correct laminar organization of cortical organoids. (Left), day-60 forebrain organoid sections generated with antioxidant (+AO) or without antioxidants (-AO+vA^{lo}), or the latter supplemented with ferrostatin-1 (-AO+vA^{lo}+Fer-1), and stained for TBR1. Quantification of TBR1+ cells using ImageJ analysis. n = 3 biologically independent replicates. CP, cortical plate; SVZ, subventricular zone; VZ, ventricular zone. (Right), distribution of TBR1+ cells illustrated using heatmaps; F = Fer-1

Supplementary Figure 4

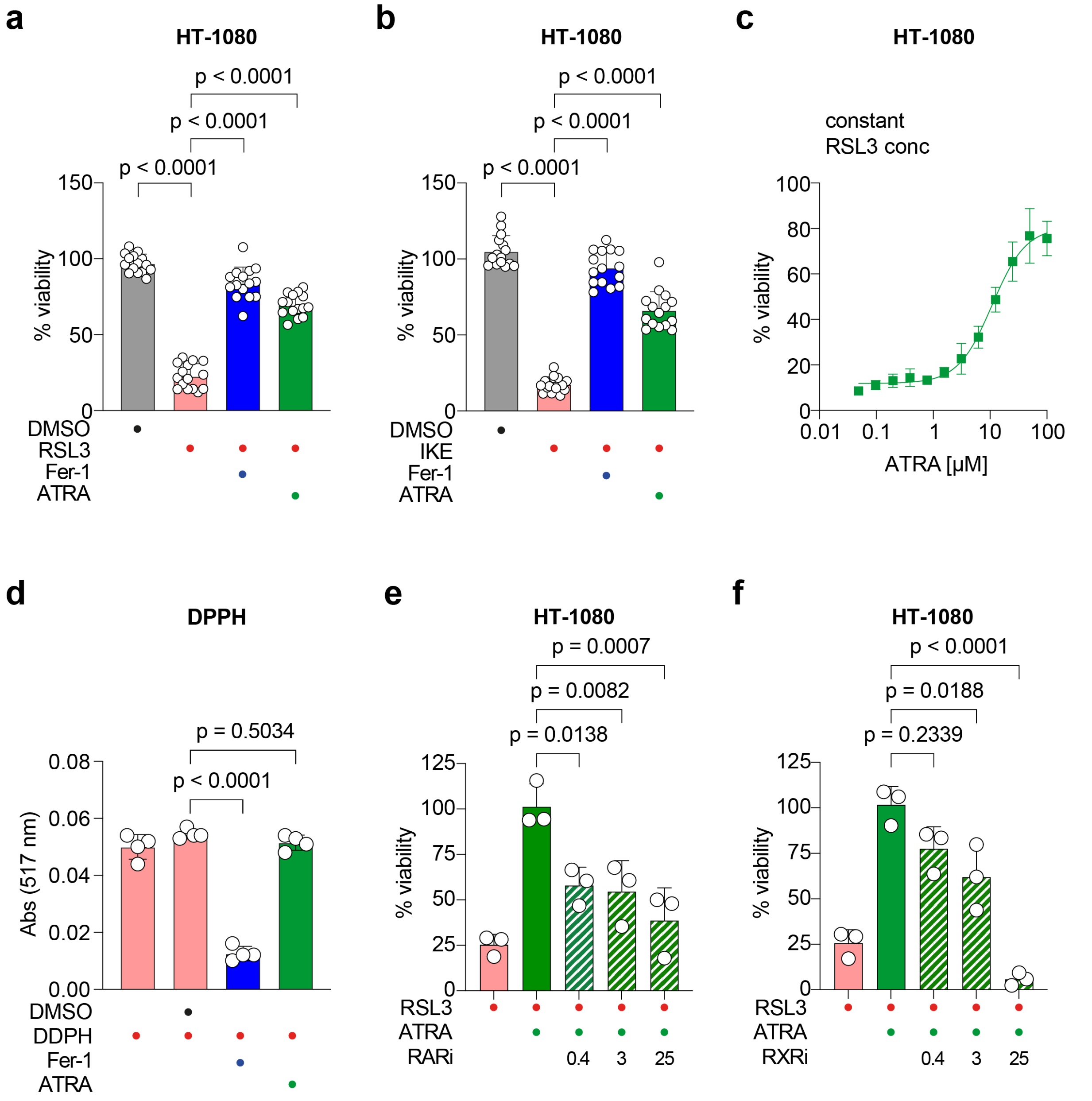

Supplementary Figure 4. Vitamin A suppresses ferroptosis in HT-1080 cells. a, CellTiter-Glo assay of HT-1080 cells co-treated with the ferroptosis inducer RSL3 and ferrostatin-1 (Fer-1) or vitamin A (ATRA). Data are mean ± SD of *n* = 15 technical replicates; one-way ANOVA with Tukey's test. **b**, CellTiter-Glo assay of HT-1080 cells co-treated with the ferroptosis inducer IKE and ferrostatin-1 (Fer-1) or vitamin A (ATRA). Data are mean ± SD of *n* = 15 technical replicates; one-way ANOVA with Tukey's test. **c**, CellTiter-Glo assay of HT-1080 cells co-treated with the ferroptosis inducer RSL3 and dose-response of vitamin A (ATRA) (*n* = 3 biological replicates). **d**, Absorbance of radical initiator 2,2-diphenyl-1-picrylhydrazyl (DPPH) co-treated with ferrostatin-1 (Fer-1) or vitamin A (ATRA). Data are mean ± SD of *n* = 4 biologically independent replicates; one-way ANOVA with Tukey's test. **e**, CellTiter-Glo assay of HT-1080 cells after co-treatment with RSL3 and vitamin A (ATRA) as well as different concentrations of the small molecule inhibitor of Retinoic Acid Receptor (RARi). Data are mean ± SD of *n* = 3 biologically independent replicates; one-way-ANOVA with Tukey's test. **f**, CellTiter-Glo assay of HT-1080 cells after co-treatment with RSL3 and vitamin A (ATRA) as well as different concentrations of the small molecule inhibitor of Retinoid X receptor (RXRi). Data are mean ± SD of *n* = 3 biologically independent replicates; one-way ANOVA with Tukey's test.

Supplementary Figure 5

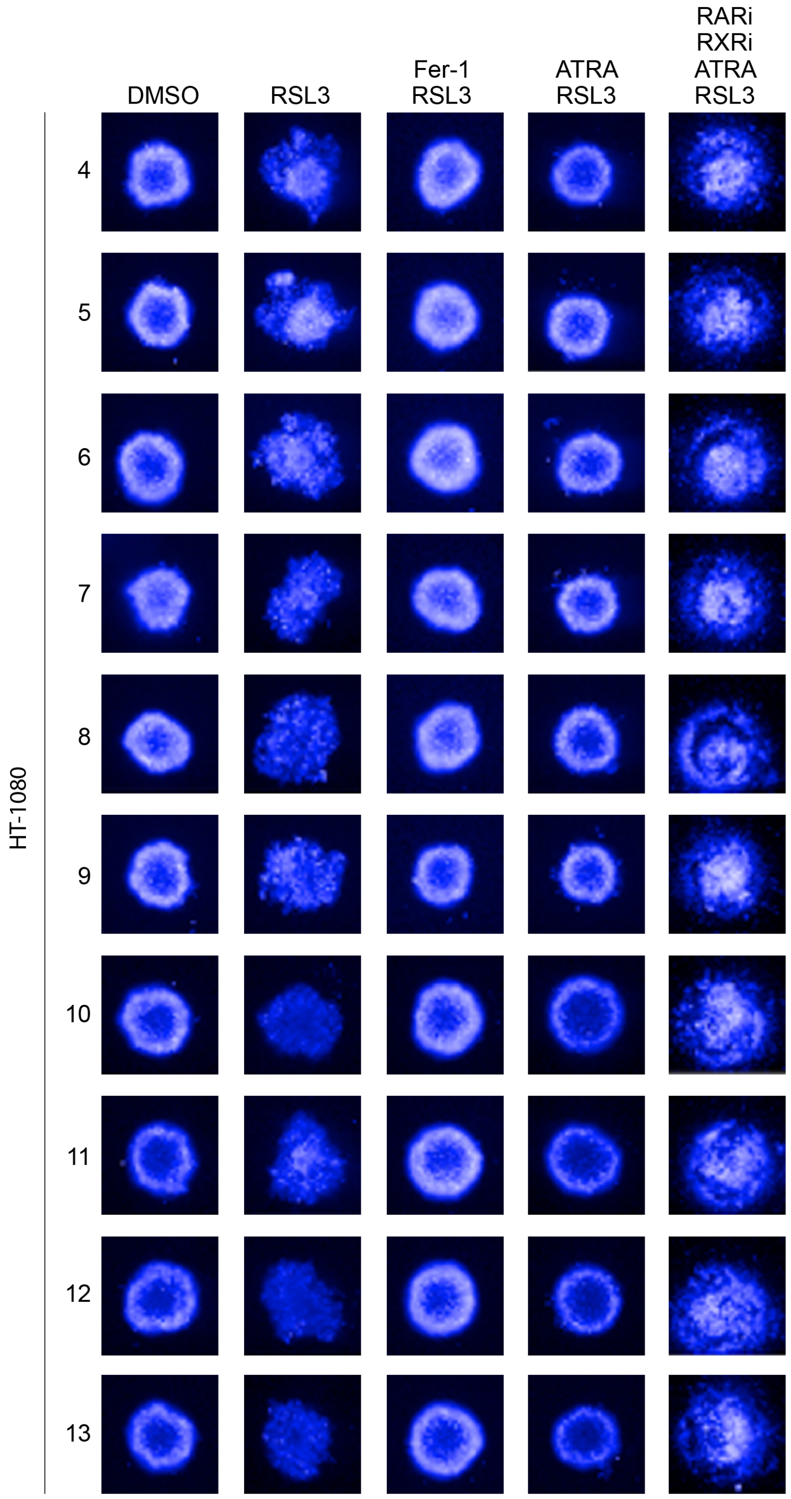

Supplementary Figure 5. Inhibition of ferroptosis by vitamin A impacts spheroid growth. Remaining replicates of spheroids with the indicated treatments corresponding to Fig. 1h.

Supplementary Figure 6

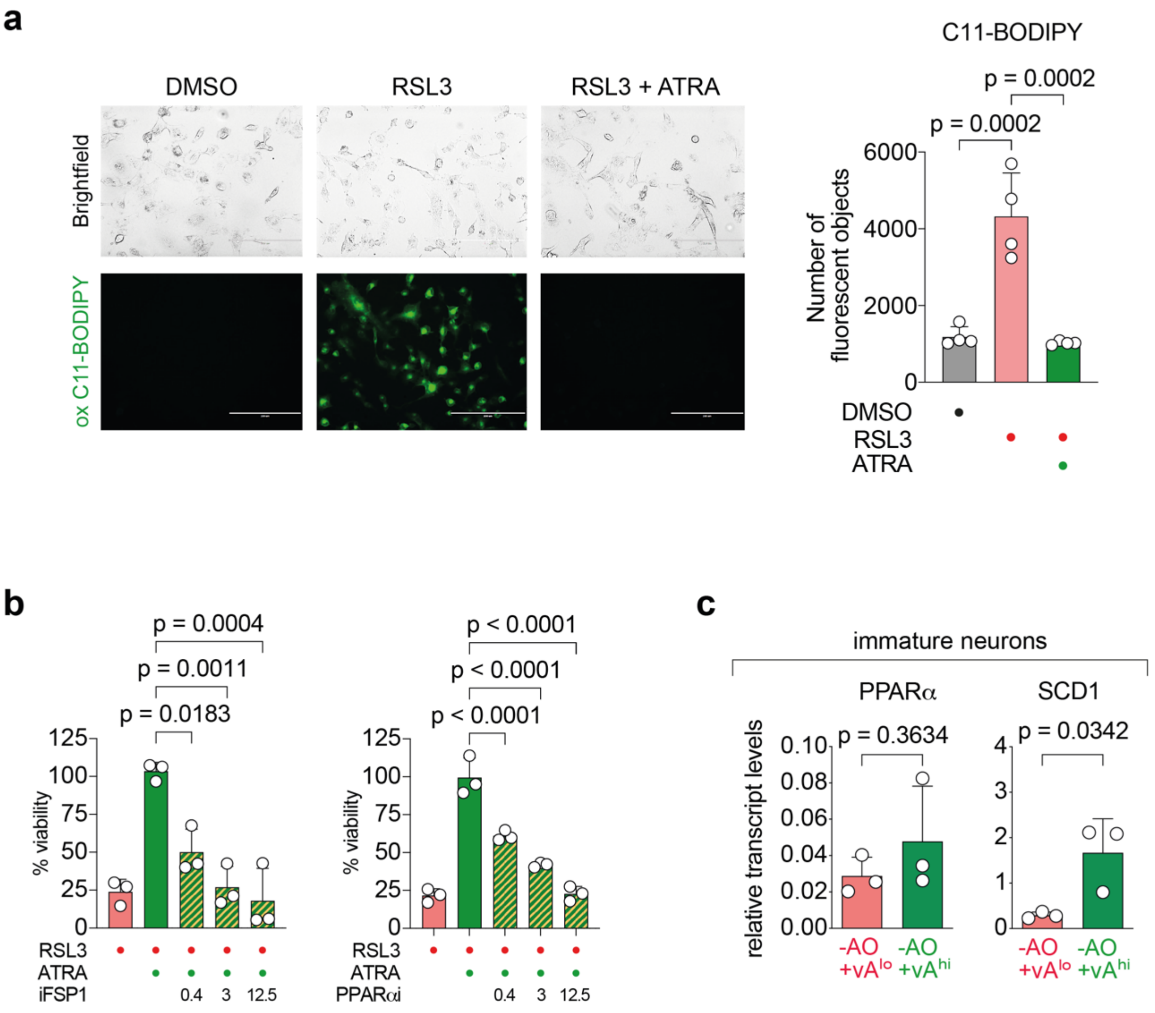

Supplementary Figure 6. Vitamin A eliminates lipid peroxidation and suppresses ferroptosis. a, (Left), C11-BODIPY microscopy images of HT-1080 cells co-treated with RSL3 and vitamin A (ATRA); (Right), Quantification of fluorescent objects. Data are mean ± SD of *n* = 4 biologically independent replicates; one-way ANOVA with Tukey's test. **b**, CellTiter-Glo assay of co-treated HT-1080 cells with RSL3, vitamin A (ATRA) and inhibitors against FSP1 (iFSP1) or PPARα (GW6471). Data are mean ± SD of *n* = 3 biologically independent replicates; one-way ANOVA with Tukey's test. **c**, Relative mRNA levels of PPARα and SCD1 in immature neurons (day 20) measured by quantitative RT-PCR. Data are mean ± SD of *n* = 3 biologically independent replicates; unpaired t-test, two-tailed.

Supplementary Figure 7

Figure 5e (HT-1080)

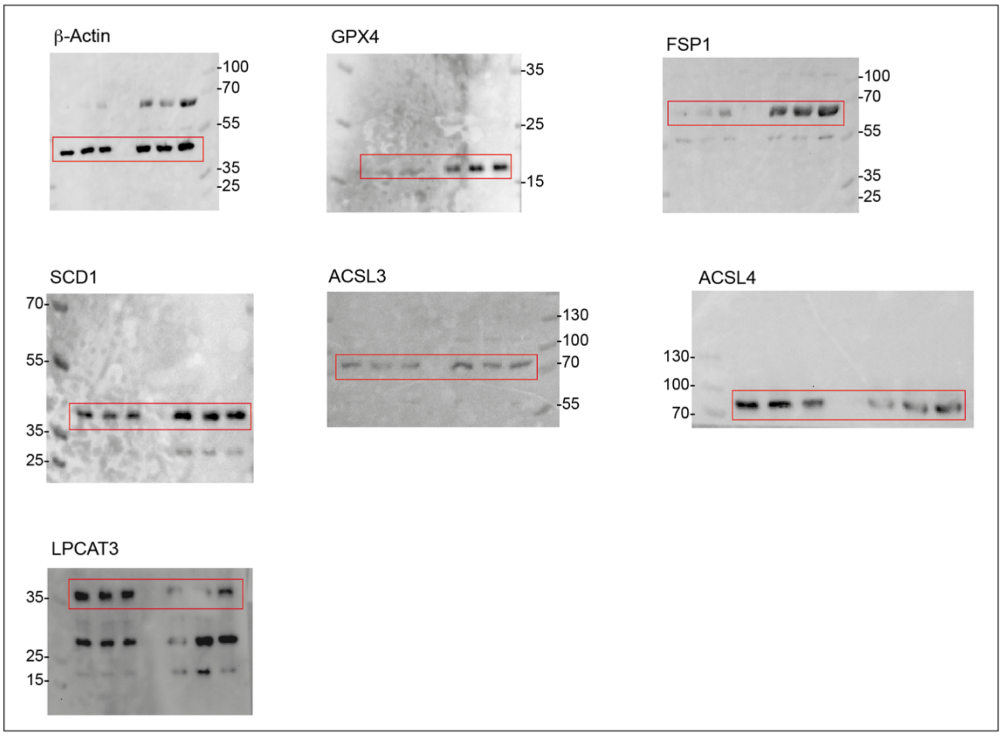

Figure 5g (immature neurons)

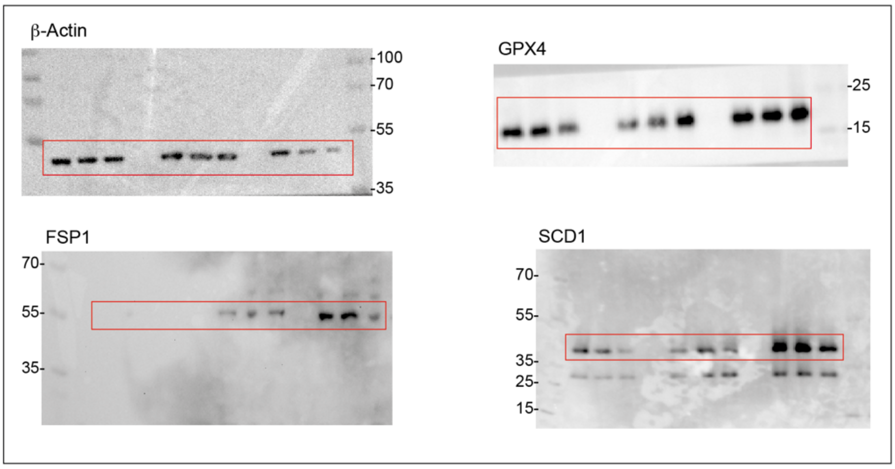

Supplementary Figure 7. Full scans of the Western Blots.
Uncropped Western Blot images of Fig. 5e and 5g.
