# Supplementary material for: Suppression of ferroptosis by vitamin A or radical-trapping antioxidants is essential for neuronal development
Source: Nat Commun. 2024 Sep 1;15:7611. doi: 10.1038/s41467-024-51996-1 (PMC11366759; doi:10.1038/s41467-024-51996-1)
Supplement: Supplementary file 3 — Description of Additional Supplementary Files [file 41467_2024_51996_MOESM3_ESM.pdf]

Description of Additional Supplementary Files

File name: Supplementary Data 1
Description: Ingredients according to "Media Formulations" on ThermoFisher Scientific Webpage.

File name: Supplementary Data 2
Description: Human qRT-PCR primers.

File name: Supplementary Data 3
Description: The RNAseq data (count data) generated in this study.
