# Supplementary material for: Suppression of ferroptosis by vitamin A or radical-trapping antioxidants is essential for neuronal development
Source: Nat Commun. 2024 Sep 1;15:7611. doi: 10.1038/s41467-024-51996-1 (PMC11366759; doi:10.1038/s41467-024-51996-1)
Supplement: Supplementary file 7 — Reporting Summary [file 41467_2024_51996_MOESM7_ESM.pdf]

nature portfolio

Corresponding author(s): Kamyar Hadian, Michelle Vincendeau

Last updated by author(s): Aug 1, 2024

Reporting Summary

Nature Portfolio wishes to improve the reproducibility of the work that we publish. This form provides structure for consistency and transparency in reporting. For further information on Nature Portfolio policies, see our Editorial Policies and the Editorial Policy Checklist.

Statistics

For all statistical analyses, confirm that the following items are present in the figure legend, table legend, main text, or Methods section.

n/a	Confirmed	
☐	☒	The exact sample size (*n*) for each experimental group/condition, given as a discrete number and unit of measurement
☐	☒	A statement on whether measurements were taken from distinct samples or whether the same sample was measured repeatedly
☐	☒	The statistical test(s) used AND whether they are one- or two-sided<br>*Only common tests should be described solely by name; describe more complex techniques in the Methods section.*
☒	☐	A description of all covariates tested
☐	☒	A description of any assumptions or corrections, such as tests of normality and adjustment for multiple comparisons
☐	☒	A full description of the statistical parameters including central tendency (e.g. means) or other basic estimates (e.g. regression coefficient) AND variation (e.g. standard deviation) or associated estimates of uncertainty (e.g. confidence intervals)
☐	☒	For null hypothesis testing, the test statistic (e.g. *F*, *t*, *r*) with confidence intervals, effect sizes, degrees of freedom and *P* value noted<br>*Give P values as exact values whenever suitable.*
☒	☐	For Bayesian analysis, information on the choice of priors and Markov chain Monte Carlo settings
☒	☐	For hierarchical and complex designs, identification of the appropriate level for tests and full reporting of outcomes
☒	☐	Estimates of effect sizes (e.g. Cohen's *d*, Pearson's *r*), indicating how they were calculated

Our web collection on statistics for biologists contains articles on many of the points above.

Software and code

Policy information about availability of computer code

Data collection	PerkinElmer EnVision 2104 Multilabel plate reader; PerkinElmer Operetta high-content imaging/screening system; Roche LightCycler 480; Applied Biosystems Attune acoustic flow cytometer; EVOS FL fluorescence microscope, Zeiss (AX10) microscope, Zeiss LSM980 microscope
Data analysis	PerkinElmer Columbus Software version 2.9.1; BD Life Sciences FlowJo v10.8.1 Software; Genedata Expressionist 13.5; R software version 4.2.0 with DESeq2 package; GraphPad Prism version 9.4

For manuscripts utilizing custom algorithms or software that are central to the research but not yet described in published literature, software must be made available to editors and reviewers. We strongly encourage code deposition in a community repository (e.g. GitHub). See the Nature Portfolio guidelines for submitting code & software for further information.

Data

Policy information about availability of data

All manuscripts must include a data availability statement. This statement should provide the following information, where applicable:

- Accession codes, unique identifiers, or web links for publicly available datasets
- A description of any restrictions on data availability
- For clinical datasets or third party data, please ensure that the statement adheres to our policy

RNAseq count table with all data are available in Supplementary Table 3. Source code of analysis of differentially expressed genes is available at https://github.com/MendenLab/DEx_StemCells_new.

Research involving human participants, their data, or biological material

Policy information about studies with human participants or human data. See also policy information about sex, gender (identity/presentation), and sexual orientation and race, ethnicity and racism.

Reporting on sex and gender	No human research participants for this study.
Reporting on race, ethnicity, or other socially relevant groupings	No human research participants for this study.
Population characteristics	no population data
Recruitment	not applicable
Ethics oversight	not applicable

Note that full information on the approval of the study protocol must also be provided in the manuscript.

Field-specific reporting

Please select the one below that is the best fit for your research. If you are not sure, read the appropriate sections before making your selection.

☒ Life sciences ☐ Behavioural & social sciences ☐ Ecological, evolutionary & environmental sciences

For a reference copy of the document with all sections, see nature.com/documents/nr-reporting-summary-flat.pdf

Life sciences study design

All studies must disclose on these points even when the disclosure is negative.

Sample size	No sample size calculations were performed. For every experiment (where applicable), at least three biological replicate experiments were tested that each consisted of several technical replicates. Exact numbers of replicates are stated in the figure legends.
Data exclusions	No data was excluded
Replication	Experiments were performed 3 times independently (exception: RNA-seq). Exact replicate numbers are stated in the figure legends. Attempts at replication were successful.
Randomization	In vitro treatments of cells and organoids were performed randomized because cells were seeded into plates with the same density across every well and were mixed thoroughly before. Treatment method was the same during every experiment, e.g. well number 1 was always DMSO control etc.
Blinding	Except for randomization, we did not actively take blinding measures in our experiments. However, we tried to avoid bias by performing experiments in several biological replicates and by not excluding important data. Experiments were performed by different researchers from collaborating groups.

Reporting for specific materials, systems and methods

We require information from authors about some types of materials, experimental systems and methods used in many studies. Here, indicate whether each material, system or method listed is relevant to your study. If you are not sure if a list item applies to your research, read the appropriate section before selecting a response.

Materials & experimental systems

n/a	Involved in the study	
☐	☒	Antibodies
☐	☒	Eukaryotic cell lines
☒	☐	Palaeontology and archaeology
☒	☐	Animals and other organisms
☒	☐	Clinical data
☒	☐	Dual use research of concern
☒	☐	Plants

Methods

n/a	Involved in the study	
☒	☐	ChIP-seq
☐	☒	Flow cytometry
☒	☐	MRI-based neuroimaging

Antibodies

Antibodies used

rabbit anti-4-Hydroxynonenal-antibody (4-HNE, ab46545, Abcam, RRID:AB_722490)
anti-rabbit Alexa 488 antibody (A32731, Thermo Fisher Scientific, RRID:AB_2633280)
mouse monoclonal Anti-MAP2 (M-1406, Sigma-Aldrich, RRID:AB_477171)
AlexaFluor Goat Anti-Mouse 488 (R37120, Thermo Fisher Scientific, RRID:AB_2556548)
Mouse monoclonal Anti-SOX2 (sc-365823, SantaCruz Biotech, RRID:AB_10842165)
Rat monoclonal Anti-CTIP2 (ab18465, Abcam, RRID:AB_2064130)
Rabbit polyclonal Anti-TBR1 (ab31940, Abcam, RRID:AB_2200219)
Rabbit polyclonal Anti-SATB2 (HPA001042, Sigma-Aldrich, RRID:AB_10601711)
Mouse Anti-TfR1 (CD71—3B8 2A1) antibody (sc-32272, Santa Cruz)
AlexaFluor Goat Anti-Rat 488 (ab150157, Abcam, RRID:AB_2722511)
AlexaFluor Goat Anti-Mouse 594 (ab150116, Abcam, RRID:AB_2650601)
AlexaFluor Goat Anti-Rabbit 594 (A11012, Thermo Fisher Scientific, RRID:AB_141359)
AlexaFluor Goat Anti-Rat 568 (A-11077, Thermo Fisher Scientific, RRID:AB_141874)
AlexaFluor Goat Anti-Rabbit 647 (A-27040, Thermo Fisher Scientific, RRID:AB_2536101)

Validation

Commercial antibodies were used, which were rigorously validated by their distributors for specificity and functionality.

Validation statements
Abcam: "Antibodies are validated in western blot using lysates from cells or tissues that we have identified to express the protein of interest. Once we have determined the right lysates to use, western blots are run and the band size is checked for the expected molecular weight. We will always run several controls in the same western blot experiment, including positive lysate and negative lysate. When possible, we also include knock-out (KO) cell lines as a true negative control for our western blots. We are always increasing the number of KO-validated antibodies we provide. In addition, we run old stock alongside our new stock. If we know the old stock works well, this also acts as a suitable positive control. If the western blot result gives a clear clean band and we are happy with the result from the control lanes, these antibodies will be passed and added to the catalog."

Thermo Fisher Scientific: "Invitrogen antibodies are currently undergoing a rigorous two-part testing approach:
Part 1—Target specificity verification

This helps ensure the antibody will bind to the correct target. Our antibodies are being tested using at least one of the following methods to ensure proper functionality in researcher's experiments. Click on each testing method below for detailed testing strategies, workflow examples, and data figure legends.

Knockout—expression testing using CRISPR-Cas9 cell models
Knockdown—expression testing using RNAi to knockdown gene of interest
Independent antibody verification (IAV)—measurement of target expression is performed using two differentially raised antibodies recognizing the same protein target
Cell treatment—detecting downstream events following cell treatment
Relative expression—using naturally occurring variable expression to confirm specificity
Neutralization—functional blocking of protein activity by antibody binding
Peptide array—using arrays to test reactivity against known protein modifications
SNAP-ChIP™—using SNAP-ChIP to test reactivity against known protein modifications
Immunoprecipitation-Mass Spectrometry (IP-MS)—testing using immunoprecipitation followed by mass spectrometry to identify antibody targets

Part 2—Functional application validation

These tests help ensure the antibody works in a particular application(s) of interest, which may include
(but are not limited to):

Western blotting
Flow cytometry
ChIP
Immunofluorescence imaging
Immunohistochemistry

Most antibodies were developed with specific applications in mind. Testing that an antibody generates acceptable results in a specific application is the second part of confirming antibody performance."

Santa Cruz Biotechnology: "Santa Cruz Biotechnology is expanding our monoclonal antibody product line. We offer monoclonal antibodies directed against a broad range of mammalian and non-mammalian protein targets, representing essentially all targets covered by polyclonal antibodies. Primary antibodies directed to mammalian target proteins have been characterized for reactivity against mouse, rat and human proteins. Many of our mammalian antibodies are reactive with equine, bovine, canine, feline, caprine, porcine and ovine protein targets and are suitable for veterinary research. Primary antibodies directed to non-mammalian target proteins, including proteins of bacterial, viral, plant, zebrafish and Drosphila origin, are available. Our antibodies are recommended for use in most assays including Western blot, immunoprecipitation, immunostaining, and flow cytometry. We also offer a wide variety of secondary antibodies, control immunoglobulin and control sera for a large selection of species."

Eukaryotic cell lines

Policy information about cell lines and Sex and Gender in Research

Cell line source(s)	HT-1080 were purchased from ATCC; H9 (WA09) were obtained from the WiCell Research Institute.
Authentication	H9 (WA09) cells were routinely authenticated by measuring levels of Nanog and Oct-4; HT-1080 were not further authenticated.
Mycoplasma contamination	HT-1080 and H9 (WA09) were regularly tested for mycoplasma contamination via PCR; the results were negative during the time of this study.
Commonly misidentified lines (See ICLAC register)	No commonly misidentified lines were used.

Flow Cytometry

Plots

Confirm that:

☒ The axis labels state the marker and fluorochrome used (e.g. CD4-FITC).

☒ The axis scales are clearly visible. Include numbers along axes only for bottom left plot of group (a 'group' is an analysis of identical markers).

☒ All plots are contour plots with outliers or pseudocolor plots.

☒ A numerical value for number of cells or percentage (with statistics) is provided.

Methodology

Sample preparation

Immunostaining of HT-1080 with anti-4-Hydroxynonenal-antibody:
Ferroptotic cell death was induced via 300 nM RSL3 for 2 h and cells were co-treated with 20 µM ATRA or 2 µM Ferrostatin-1. 10% normal goat serum (Thermo Fisher Scientific) was used for blocking before cells were incubated in anti-4-HNE antibody (1:50 in 1% BSA in PBS, ab46545, Abcam). As a secondary antibody, anti-rabbit Alexa 488 antibody (1:200 in 1% BSA in PBS, A32731, Thermo Fisher Scientific) was used. For flow cytometry, 10,000 events per condition were measured in the BL-1 channel of an Attune acoustic flow cytometer (Applied Biosystems).

Immunostaining of H9 differentiated into neurons with anti-4-Hydroxynonenal-antibody:
After differentiation for 40 days, cells were harvested and 10% normal goat serum (Thermo Fisher Scientific) was used for blocking before cells were incubated in anti-4-HNE antibody (1:50 in 1% BSA in PBS, ab46545, Abcam)). As a secondary antibody, anti-rabbit Alexa 488 antibody (1:200 in 1% BSA in PBS, A32731, Thermo Fisher Scientific) was used. For flow cytometry, 10,000 events per condition were measured in the BL-1 channel of an Attune acoustic flow cytometer (Applied Biosystems).

Immunostaining of forebrain organoids with anti-4-Hydroxynonenal-antibody:
After differentiation for 40 days, organoids were dissociated, harvested and 10% normal goat serum (Thermo Fisher Scientific) was used for blocking before cells were incubated in anti-4-HNE antibody (1:50 in 1% BSA in PBS, ab46545, Abcam)). As a secondary antibody, anti-rabbit Alexa 488 antibody (1:200 in 1% BSA in PBS, A32731, Thermo Fisher Scientific) was used. For flow cytometry, 10,000 events per condition were measured in the BL-1 channel of an Attune acoustic flow cytometer (Applied Biosystems).

C11-BODIPY staining of HT-1080 and H9 differentiated into neurons:
Ferroptosis was induced for 2 h with 250 nM RSL3 and cells were co-treated with 20 µM ATRA or 2 µM Ferrostatin-1. HT-1080 cells were stained with 2 µM C11-BODIPY for 30 min. Day-20 neurons were stained with 2 µM C11-BODIPY for 1 h. Afterwards cells were measured in the BL-1 channel of an Attune acoustic flow cytometer (Applied Biosystems).

Instrument	Applied Biosystems Attune acoustic flow cytometer
Software	BD Life Sciences FlowJo v10.8.1 Software
Cell population abundance	No sorting was performed. Flow cytometry was used for detection of lipid peroxidation and ferroptosis via C11-BODIPY and 4-HNE staining.
Gating strategy	FSC/SSC gates were set to define workable cell population (live cells). Remaining population was used to generate histograms of the BL-1 channel for the different treatments. Median intensities of histograms of three replicate experiments were determined and plotted as bar graphs to evaluate statistics.

☒ Tick this box to confirm that a figure exemplifying the gating strategy is provided in the Supplementary Information.
